# Simulated Moving Bed Process for CO_2_ Capture from Humid Postcombustion Flue Gases Using MUF-16

**DOI:** 10.1021/acsami.5c16139

**Published:** 2025-10-29

**Authors:** Akriti Sarswat, Yoseph A. Guta, Mario Zorrilla-Valtierra, Anthony Cochran, Suhyun Kim, David S. Sholl, Ryan P. Lively

**Affiliations:** † School of Chemical & Biomolecular Engineering, 1372 Georgia Institute of Technology, Atlanta, Georgia 30332-0100, United States; ‡ 6146 Oak Ridge National Laboratory, Oak Ridge, Tennessee 37830, United States

**Keywords:** simulated moving bed, postcombustion capture of CO_2_, metal−organic frameworks, acid gas stability in MOFs, CO_2_ capture from humid flue gas

## Abstract

Simulated moving bed (SMB) designs are increasingly being adapted for the separation of multicomponent gaseous mixtures. In this work, we develop a modified SMB process for capturing CO_2_ from humid postcombustion flue gas using MUF-16 (MUF = Massey University Framework). MUF-16 shows excellent selectivity for CO_2_ over N_2_, moderate heats of adsorption for CO_2_ and H_2_O, and no competitive sorption of CO_2_ over water at relative humidities relevant to postcombustion capture. We utilize these characteristics to propose a continuous process that uses N_2_ from the feed as the desorbent for the water, eliminating the need for a separate desiccant bed and allowing for localized heating during desorption for H_2_O-saturated MOF beds. Single-component isotherms, single-column breakthrough experiments, and SO_2_ stability tests suggest the excellent suitability of MUF-16 to this separation via the proposed SMB design.

## Graphical abstract

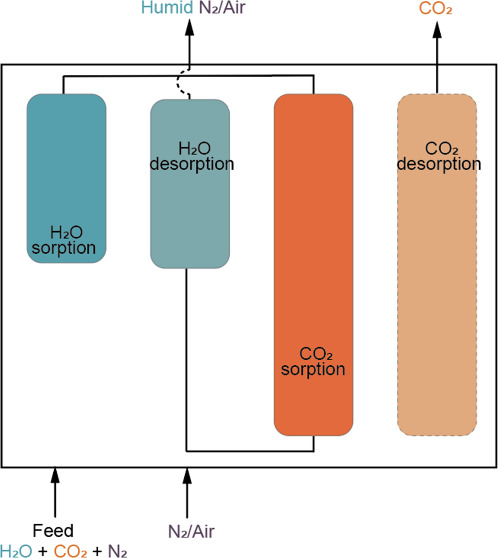

## 1 Introduction

Postcombustion capture of carbon dioxide from flue gas is an essential pathway for achieving emission reduction targets, particularly via retrofitting in existing fossil fuel-based refineries.^1–4^ Currently, the overall costs associated with capturing CO_2_ from flue gas remain high, ranging between $50−100/tonne as estimated by the International Energy Agency (IEA).^5^ Extensive research has focused on developing materials and processes for this separation using absorption (e.g., in aqueous amines), which suffer from oxidative degradation of solvents, high regeneration energy requirements, and equipment corrosion.^2–4,6–8^ Consequently, there is a growing interest in developing solid sorbents as alternatives.^2–4,9^ Among these, metal–organic frameworks (MOFs), due to their wide chemical diversity, are being extensively examined. Practical adsorbents for postcombustion CO_2_ capture should ideally have high CO_2_ working capacity in humid feeds, strong selectivity for CO_2_ over N_2_, fast adsorption–desorption kinetics, low heats of adsorption, and excellent stability in the presence of contaminants such as SO_*x*_, NO_*x*_, metal ions, etc.^10–16^

Development of effective adsorption-based CO_2_ capture processes must consider not only the adsorbent material but also the process configuration. Multiple bed configurations have been explored, including the conventional fixed bed, moving beds, fluidized beds, and simulated moving beds.^4,17–22^ The simulated moving bed (SMB) technology, first commercially introduced for *p*-xylene recovery using the Parex process, has found extensive applications in chromatography and liquid separations.^23–26^ The SMB process has also been customized to lead to various nonconventional operation modes for different applications.^27–32^ Increasingly, SMB processes have been developed to achieve separations in the gaseous phase, primarily for binary component mixtures.^33–39^ In this article, we show how an SMB process could potentially be used to take advantage of performance characteristics of a robust MOF adsorbent, leading to the possibility of a favorable CO_2_ capture process.

In this paper, we use experimental measurements and process considerations to examine the applicability of MUF-16 (MUF = Massey University Framework) to postcombustion capture. MUF-16, originally reported by Qazvini et al., shows excellent selectivity for CO_2_ in CO_2_/X mixtures (X = hydrocarbons or N_2_).^40–42^ This MOF has a low heat of adsorption for CO_2_ (approximately 36 kJ/mol) and a moderate working capacity. Qazvini et al. have demonstrated that MUF-16 has excellent kinetics of sorption and stability under humidity for more than 100 cycles of breakthrough operation.^42^ In this work, we propose a modified SMB process flow with the aim of repurposing the N_2_ raffinate from a flue gas feed stream as a desorbent for water while eliminating the need for an additional desiccant bed upstream of these adsorbers and enabling steady-state operation for this material. This design is informed by our experimental measurements of the competitive sorption of CO_2_ and H_2_O in MUF-16 and their desorption kinetics under various regeneration conditions.

While evaluating the applicability of materials for postcombustion capture, it is also vital to assess their stability upon exposure to acid gases, which are ubiquitous in flue gas streams.^43–52^ Previous reports, though limited in number, demonstrate that most MOFs tested, including MIL-101, UiO-66, and ZIF-8, show some signs of degradation upon exposure to acid gases, especially in humid streams.^46,50,52,53^ In this work, we also assess the stability of MUF-16 on exposure to dry and humid environments of SO_2_ for long periods.

## 2 Methods

### 2.1 Materials and Reactors

Cobalt­(II) acetate tetrahydrate (Alfa Aesar, 98.0–102.0%), 5-aminoisophthalic acid (Thermo Scientific, 98%), manganese­(II) acetate (Sigma-Aldrich, 98%), methanol (HPLC grade, ≥99.9%), and water (HPLC grade, ≥99.9%) were used without further purification. Reactor vessels (45 mL, part 4744) were purchased from Parr Instrument Company, USA. A gas cylinder containing 14% CO_2_, 2% He, and balance N_2_ was procured from Airgas and used as our model mixture throughout this work. For humid SO_2_ exposure, a gas cylinder containing 1000 ppm of SO_2_ (balance N_2_) was procured from Airgas. 200 ppm of the SO_2_ (balance N_2_) gas cylinder for dry SO_2_ experiments was procured from Matheson.

### 2.2 Synthesis

MUF-16 is a Co-based MOF. MUF-16 was synthesized following procedures from our previous work, where we reported that adding ppm levels of Mn to the solution mixture of the MOF, which otherwise does not contain this element, led to consistently reproducible synthesis and high-quality crystals.^41^ Cobalt­(II) acetate tetrahydrate (0.2083 g, 0.84 mmol) and 5-aminoisophthalic acid (0.6 g, 3.33 mmol) were mixed with methanol (26.67 mL) in a 45 mL Teflon-lined acid digestion pressure vessel. Separately, 20 mg of manganese­(II) acetate was added to 100 mL of water to make a stock solution. Next, 1.67 mL of this solution was added to the reaction mixture, which was then ultrasonicated for 20 min and allowed to react under autogenous pressure at 85 °C for 2 h in a preheated oven. The resulting crystals were washed with MeOH at least three times for 12 h and then activated at 150 °C under dynamic vacuum.

### 2.3 Powder Characterization

Samples were gold-sputtered using a Hummer 6 Sputterer and observed with Hitachi 8320 field emission scanning electron microscopy. Powder X-ray diffraction patterns were measured using a Rigaku Miniflex PXRD instrument between 2θ values of 4 and 60° at 5°/min and a step size of 0.01°. Cu Kα X-rays with a wavelength of 1.5406 Å were used. Our previous work demonstrated that SEM and PXRD characterizations alone were not sufficient to determine the quality of crystal samples for this material.^41^ Hence, ATR-FTIR was recorded ex situ using a Thermo Scientific Nicolet iS50 FTIR equipped with an iS50 ATR module between wavenumbers of 600 and 4000 cm^–1^. The background spectrum was characterized using dry KBr.

### 2.4 Single-Component Isotherms (CO_2_, N_2_, H_2_O)

Single-component isotherms for gaseous adsorbates were measured on a commercial volumetric adsorption analyzer, Micromeritics ASAP (Accelerated Surface Area and Porosity) 2020 Plus. Samples were activated in situ at 150 °C for at least 12 h under dynamic vacuum (<10 μmHg pressure). Uptake at each pressure point was allowed to equilibrate until the rate of change of pressure during the equilibration interval measured below 0.01% of the average pressure within that interval. H_2_O vapor isotherms were measured using commercial gravimetric sorption equipment, TA Instruments VTI-SA+. Samples were activated in situ at 150 °C for at least 12 h under nitrogen flow. At each relative humidity, uptake measurements were considered equilibrated when the change in weight over a 5 min interval was less than 0.0001% of the sample weight.

### 2.5 Breakthrough Experiments

Breakthrough experiments were conducted on a custom-built setup (SI, Figure S1). To prepare the column, 143 mg of the powder sample was packed in 14″ stainless steel tubing secured with quartz wool. Mass flows of gases were controlled by using mass flow controllers (MFCs) purchased from Alicat Scientific. Humid gas streams were generated using bubblers (250 mL gas washing bottle, Avantor #7164-16), and the relative humidity of the feed stream was controlled by adjusting the relative flow rate of dry and humid streams. Temperatures were controlled using heating tapes, and PID controllers were purchased from Omega Engineering. Gas concentrations downstream were measured using a Pfeiffer Omnistart GSD320 mass spectrometer.

### 2.6 Dry SO_2_ and CO_2_ Adsorption Measurements

CO_2_ and SO_2_ adsorption measurements were conducted by using a TA Instruments Q500 thermogravimetric analyzer. Flowing 14% CO_2_ (2% He, balance N_2_) and 200 ppm of SO_2_ in N_2_ concentrations were used. Samples were active under N_2_ flow at 150 °C for 3 h followed by 12 h adsorption for both CO_2_ and SO_2_. For cyclic measurements, the powder samples were initially activated under N_2_ flow at 150 °C for 3 h after which CO_2_ and SO_2_ uptakes were measured alternatingly for 30 min each (flow rate of 90 sccm), allowing for a desorption step after each adsorption measurement.

### 2.7 In Situ Diffuse Reflectance Infrared Fourier Transform Spectroscopy (DRIFTS)

In situ SO_2_ DRIFTS experiments were measured at 30 °C using a Thermo Scientific Nicolet iS50 FTIR instrument equipped with a Harrick Scientific Praying Mantis diffuse reflection accessory. The samples were activated in situ under 100 sccm nitrogen flow at 150 °C for 3 h followed by exposure to 1000 ppm of SO_2_ (100 sccm) while continuously collecting IR spectra until no more changes were observed.

### 2.8 Humid SO_2_ Exposure

Dry MOF samples were exposed to humid SO_2_ in a custom-built exposure setup (SI, Figure S2) for 16 h.^44,50,54,55^ The gas stream was generated by mixing 150 sccm of N_2_ and 50 sccm of SO_2_ (1000 ppm cylinder) to obtain 250 ppm overall concentration using MFCs purchased from Alicat Scientific. The nitrogen stream was flown through a bubbler for humidification to obtain a net RH of 75%.

## 3 Results and Discussion

MUF-16 was synthesized according to the procedure described in Section 2.2, yielding pink crystals. The crystals were characterized to indicate successful synthesis, as shown in SI, Section 3. Figure 1a shows the single-component volumetric CO_2_ isotherms for MUF-16 at 3 different temperatures. Significant amounts of CO_2_ are adsorbed in the MOF, with an uptake of ∼2 mmol/g at 30 °C and 100 kPa. Almost no nitrogen is absorbed at the same temperature. The isotherms are consistent with the values reported for these gases in the literature.^40–42^ Figure 1b shows gravimetric water isotherms for MUF-16 at 3 different temperatures. N_2_, which shows almost negligible adsorption in the MOF (as shown in Figure 1a), was used as a carrier gas for the experiments. These isotherms were fitted to isotherm models (SI, Section 4), and these fitted isotherms were used to obtain heats of adsorption for CO_2_ and H_2_O using the Clausius–Clapeyron equation. The average heats of adsorption over the loading ranges given in Figure 1 are 36 kJ/mol for CO_2_ and 54 kJ/mol for H_2_O. These values lie at the lower end of the typical values for such materials.^3,7^ Below, we aim to leverage these modest heats of adsorption to propose an energy-efficient flue gas separation process.

**Figure 1:**
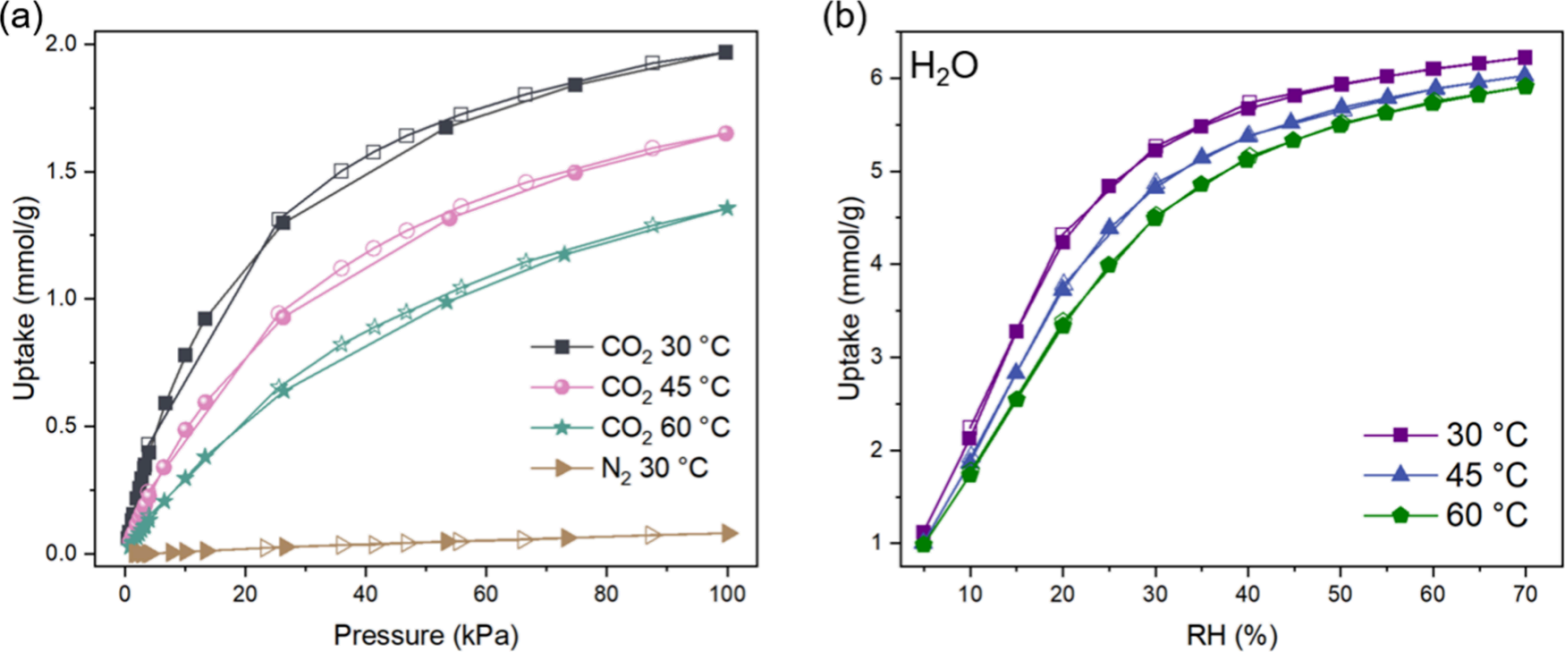
(a) Single-component CO_2_ isotherms for MUF-16 measured at 30 (black), 45 (green), and 60 °C (pink) and N_2_ isotherms measured at 30 °C (brown) and (b) H_2_O isotherms at 30 (purple), 45 (blue), and 60 °C (green) for relative humidities between 5 and 70% RH. Open symbols show loadings measured during desorption.

Figure 2 shows a schematic of our proposed SMB process for postcombustion CO_2_ capture using MUF-16. The separation of a humid flue gas mixture presents an interesting problem for the application of the SMB concept. It involves a three-component mixture (CO_2_, N_2_, and H_2_O), unlike the more common binary mixtures addressed in SMB processes.^23,56,57^ Moreover, the primary objective here is maximizing the purity and recovery of only one of the three components, namely, CO_2_. As is the case for most adsorbents, the relative binding affinity of MUF-16 follows the order H_2_O > CO_2_ > N_2_ at humidity levels relevant to flue gases. This situation poses challenges for conventional fixed bed processes because as water progressively saturates the bed it displaces CO_2_, reducing its recovery while also desorbing along with CO_2_ in some amount, affecting the purity of recovered CO_2_. A fixed bed configuration in this situation also requires frequent regeneration steps.

**Figure 2:**
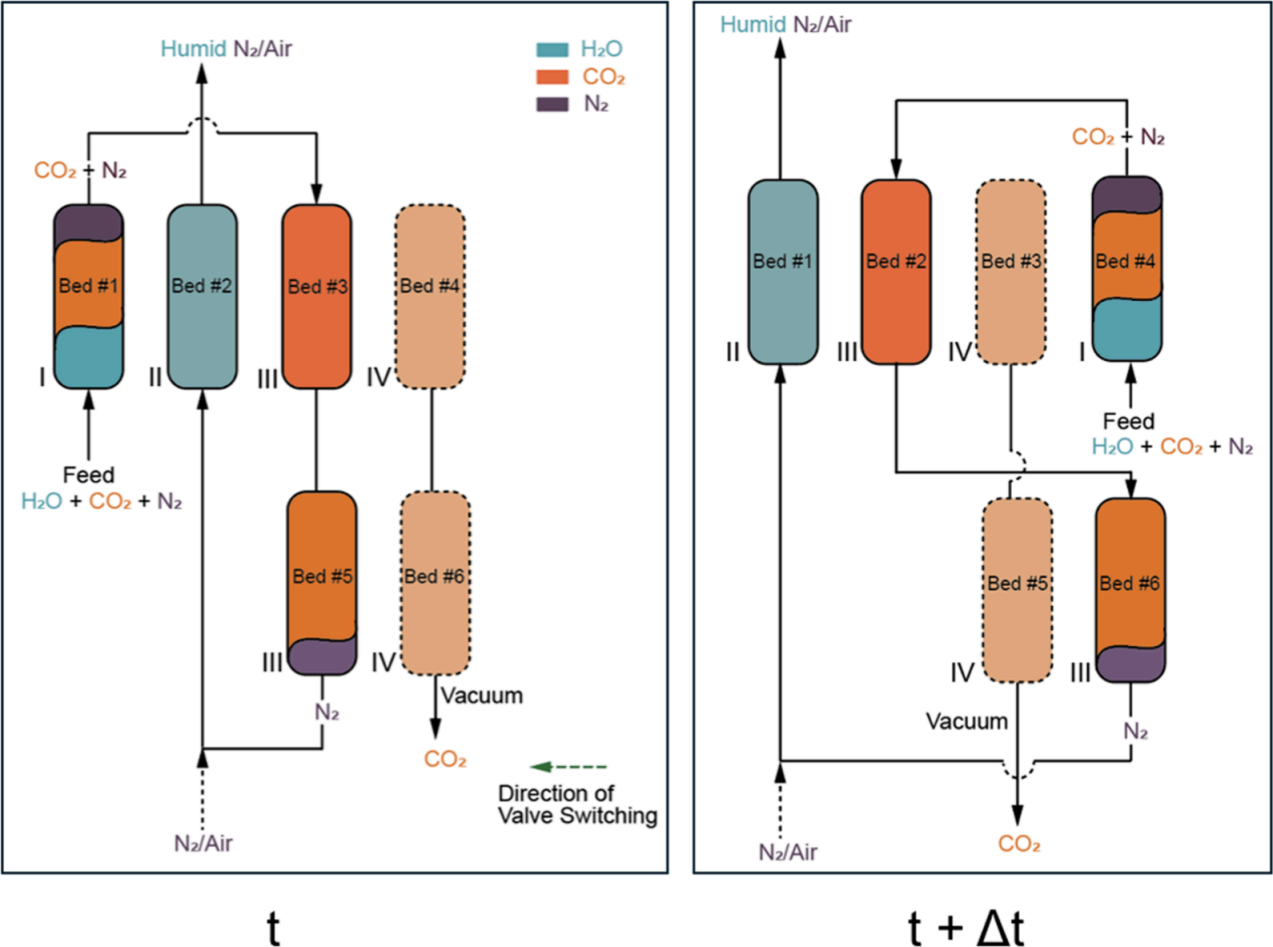
Overall schematic of the proposed SMB design at time **t** and time **t + Δt**. The beds stay fixed, while the ports attached to them change on valve switching, moving the zones of the process with the switch. Zones of operation are labeled in Roman numerals.

Our motivation for examining an SMB approach is to utilize the nitrogen raffinate for desorption of water to save on energy costs while still operating the overall process in a pseudosteady state. As shown in Figure 2, at time **t**, the flue gas is fed to bed #1 where water is adsorbed, while CO_2_ and N_2_ exit the bed as the weakly adsorbing components. This is zone I of operation. The effluent mixture of CO_2_ and N_2_ needs to be separated by adsorbing CO_2_ in zone III. Due to the different capacities of the adsorbent for CO_2_ and H_2_O at the flue gas concentrations, steady-state operation requires a longer bed in zone III than in zone I. In an SMB design, however, all beds in the cycle must be of the same size as they each progressively loop through all stages. For this reason, bed #5 and bed #6 are used as auxiliary beds to increase the column length in zone III. Therefore, at time **t**, a N_2_+CO_2_ mixture is fed to bed #3, which is in turn connected to bed #5 to obtain N_2_ as the raffinate. N_2_ is then mixed with air (which has a lower relative humidity than flue gas) or additional nitrogen, as desired, to use this mixture as a desorbent for regenerating the water in bed #2 that had previously adsorbed in zone I of the previous time step to obtain a humid desorbent stream (zone II). A fraction of this desorbent stream can be stripped of water and recycled back as a desorbent, while the rest can simply be vented. Crucially, the dry N_2_ stream obtained from bed #3 reduces the amount of desorbent stream that needs to be dried, reducing the total energy input for the process. In beds #4 and #6, CO_2_ desorption takes place at time **t** under vacuum (zone IV). Beds #4 and #6 had absorbed CO_2_ in the previous time step (zone III).

After an interval of Δ*t*, the valves (and by extension, zones within the SMB) switch toward the left (right panel, Figure 2). Bed #1, which was adsorbing water at time **t**, now enters zone II, which receives the desorbent for its regeneration. Beds #2 and #6, now in zone III, were prepared for CO_2_/N_2_ separation in the previous step. Beds #3 and #5, which were earlier adsorbing CO_2_, are exposed to a vacuum to recover CO_2_ (zone 4). Finally, bed #4, which was earlier a part of zone IV, is moved to zone I after being prepared to receive humid streams by desorption of CO_2_ under a vacuum. Overall, beds #1–4 go through all zones over four time steps, while auxiliary beds #5 and #6 that assist in altering effective bed length in zone III successively switch between adsorbing (zone III) and desorbing CO_2_ (zone IV).

To demonstrate that the SMB process described conceptually above is actually feasible with MUF-16, it is important to establish a variety of performance characteristics experimentally. For example, the time period of switching valves (Δ*t*), desorption conditions for water and CO_2_, and the relative flow rates of feed and additional N_2_/air input need to be decided. This information cannot reliably be determined from single-component isotherms such as those shown above. Hence, we performed a variety of single-column breakthroughs with gas mixtures and derived our proposed process parameters from those results.

To evaluate competitive sorption of H_2_O and CO_2_ in MUF-16, we conducted breakthrough experiments as described in Section 2.5. In these experiments, the bed was presaturated with water vapor by flowing humidified nitrogen at different relative humidities (50 and 70%) at 30 °C. After water breakthrough was achieved for a given RH, a 14% CO_2_ (2% He, balance N_2_) feed stream at the same humidity was introduced to the bed. As shown in Figure 3, in both cases, the He tracer and CO_2_ break through at essentially the same time with sharp fronts, demonstrating that in the presence of water at these RH% there is no coadsorption of CO_2_ with water. While CO_2_ and H_2_O coadsorption can be advantageous in some processes, in this case, in our proposed SMB process, the lack of coadsorption is beneficial as it leads to sharp water and CO_2_ fronts in zone I, minimizing CO_2_ residues in the bed in zone I and providing a higher CO_2_ concentration feed to zone III.^3,7,16^ We observed a minor roll-up of CO_2_ upon breakthrough, which may be attributed to pressure and compositional recalibration in the bed upon switching to feed flow. This speculation is supported by the lack of an observed roll-up at lower flow rates of the feed (SI, Section 5).

**Figure 3:**
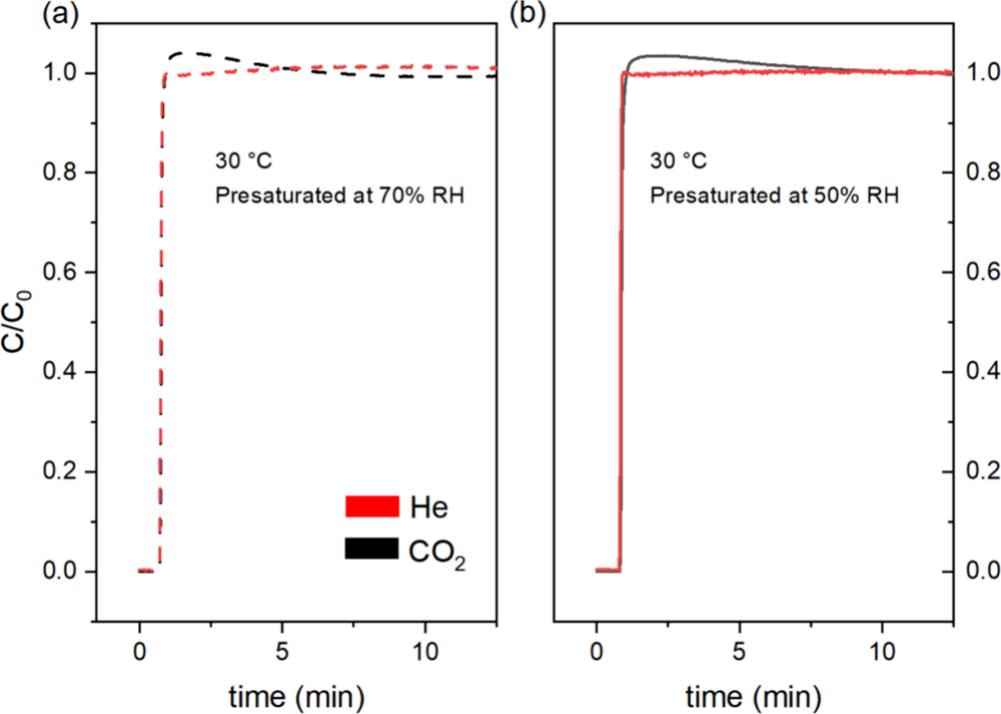
Experimental breakthrough curves for CO_2_ (black) and He (red) when fed to the powder bed presaturated at (a) 70% and (b) 50% relative humidities at 30 °C. The feed consisted of 14% CO_2_, 2% He, and balance N_2_.

In our proposed model, the feed (zone I) always sees a dry bed prepared for adsorption in zone IV. For this reason, the remainder of our breakthrough experiments were conducted on dry activated samples with no presaturation. All experiments used the same module as the previous experiments (i.e., the same MOF sample and hence the same weight of the sample in the bed). Figure 4a shows the breakthrough curves on feeding the dry CO_2_/He/N_2_ (14%/2%/ 84%) feed to the bed at 30 °C. Helium was used as a tracer, and the breakthrough profile of He captures the dynamics of non-adsorbing gases in the column. It must be noted that *t* = 0 in all of the reported breakthrough profiles is an arbitrary reference point. The absolute value of the time of breakthrough of He in these curves is confounded with delays associated with onset of data recording, valve switching, and time offset applied for clarity in graphs. The premixed cylinder contains He as the tracer so the start of the experiment can be unambiguously represented in the data via the helium breakthrough. Some helium roll-up is observed in the breakthrough experiments due to displacement of He from voids in the bed as the CO_2_ front progresses. Total uptake amounts obtained by integrating the area under the breakthrough curve are consistent with the uptakes obtained from single-component isotherm experiments (SI, Section 5).

**Figure 4:**
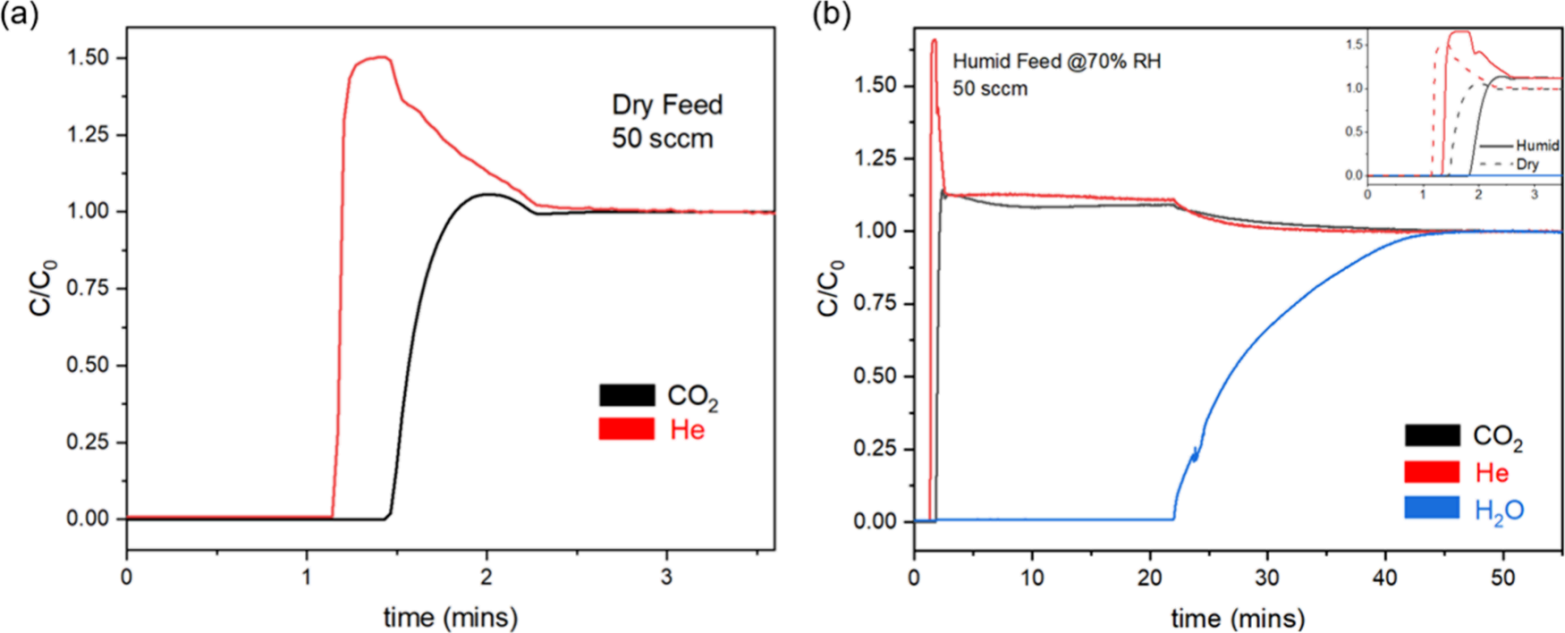
(a) CO_2_ breakthrough curves for MUF-16 with a dry 14% CO_2_ feed at 30 °C. He (2%) was used as a tracer. Breakthrough of He indicated onset of adsorption. (b) CO_2_ breakthrough curves for a dry MUF-16 bed with the 14% CO_2_ feed at 70% RH, 30 °C. The inset shows the CO_2_ profile for both dry (same as (a), dashed) and humid (solid) feeds.

Figure 4b shows a breakthrough curve for the feed stream (14% CO_2_/2%He/84% N_2_) at 70% relative humidity and 30 °C over a dry bed. Water was observed to require more than 20 min to break through, while for the relatively weakly adsorbing species (He, CO_2_, and N_2_), breakthrough occurred in less than a minute at the given mass of samples and flow rates. This observation justifies the need for auxiliary beds in the proposed SMB process. The process ideally requires valve switching just before water breaks through zone I, which should coincide with the time preceding the breakthrough of CO_2_ in zone III for maximum bed utilization and CO_2_ recovery. To facilitate this coordination, zone III is supplied with the auxiliary bed, which provides a bed for CO_2_ adsorption in the zone while water saturates the bed in zone I. This potentially leads to the continuous production of N_2_ raffinate, which can then be used as a desorbent in zone II at the expense of increased capital costs.

Prior to the valve switch, the beds in the two zones undergoing desorption of CO_2_ and H_2_O must be regenerated completely and cooled down, if heat was used, to be ready for the next adsorption step. Figure 5 shows desorption kinetics of CO_2_ under different nitrogen flow rates after adsorption of the 14% CO_2_ feed in the bed (50 sccm, 30 °C, as in Figure 4a) without any additional heating, i.e., at the adsorption temperature of 30 °C. The helium tracer concentration dropping to zero indicates the onset of desorption. In all cases, CO_2_ is readily desorbed from the bed in 5 min or less for our module. Once the outlet concentration dropped to zero, the bed was heated to 110 °C to confirm complete desorption of CO_2_. We see that in each case no further CO_2_ was desorbed, indicating complete desorption without requiring heat. In the proposed SMB process, vacuum could readily be used for CO_2_ desorption, but desorption of CO_2_ is expected to be quick and does not constrain the choice of process parameters.

**Figure 5:**
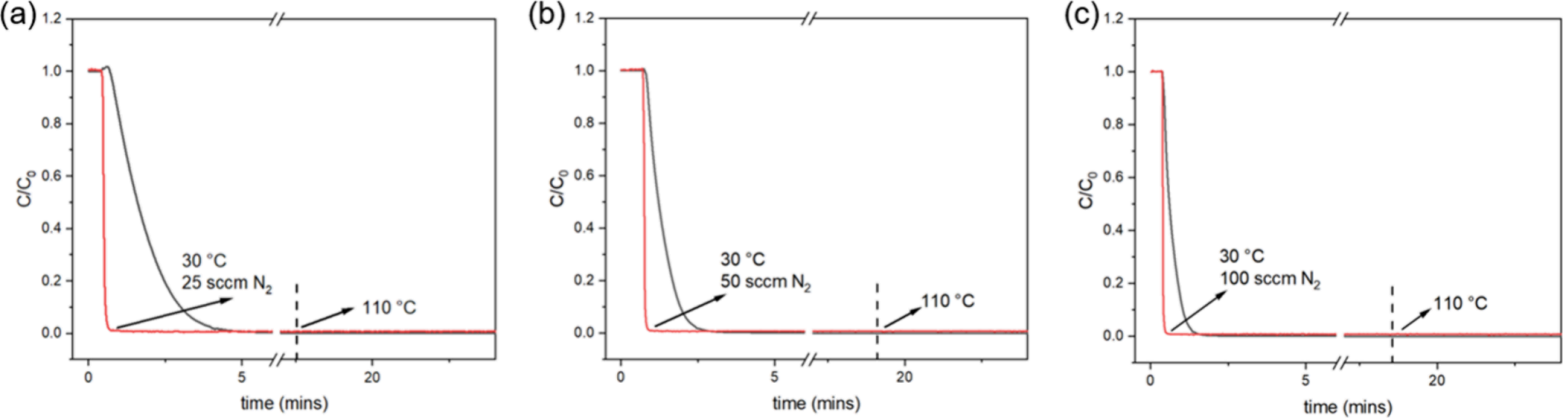
Desorption profiles of CO_2_ after adsorption of dry simulated flue gas (14% CO_2_, 30 °C) in a breakthrough experiment at 30 °C using (a) 25 sccm N_2_, (b) 50 sccm N_2_, and (c) 100 sccm N_2_. Dashed lines indicate the onset of heating the module, where the temperature is set to 110 °C.

To determine water desorption conditions for zone II, desorption kinetics were measured with different flow rates and temperatures of the desorbent (N_2_ or air). Figure 6 shows water desorption from the humid breakthrough experiments. For each of these experiments, water was allowed to break through (as in Figure 4b) before switching to desorption conditions (as in Figure 4b). In each case, no CO_2_ desorption occurs, reinforcing the conclusions from Figure 3 that there is no coadsorption of CO_2_ and H_2_O. Figure 6a shows that with a desorbent flow rate of 100 sccm at the adsorption temperature of 30 °C complete water desorption can be achieved in about 1 h with no additional heating. Complete desorption with these conditions is validated by the fact that no additional desorption of water occurs upon heating to 110 °C. Heating the bed to just 45 °C leads to a reduction in desorption time by ∼25%, as shown in Figure 6b. Heating to 60 °C cuts the time roughly by half compared to desorption at 30 °C. When a desorbent flow rate of 50 sccm is used (Figure 6d), the total time approximately doubles for our bed configuration. Additionally, in this case, the desorption is incomplete as some residual water is released upon heating.

**Figure 6:**
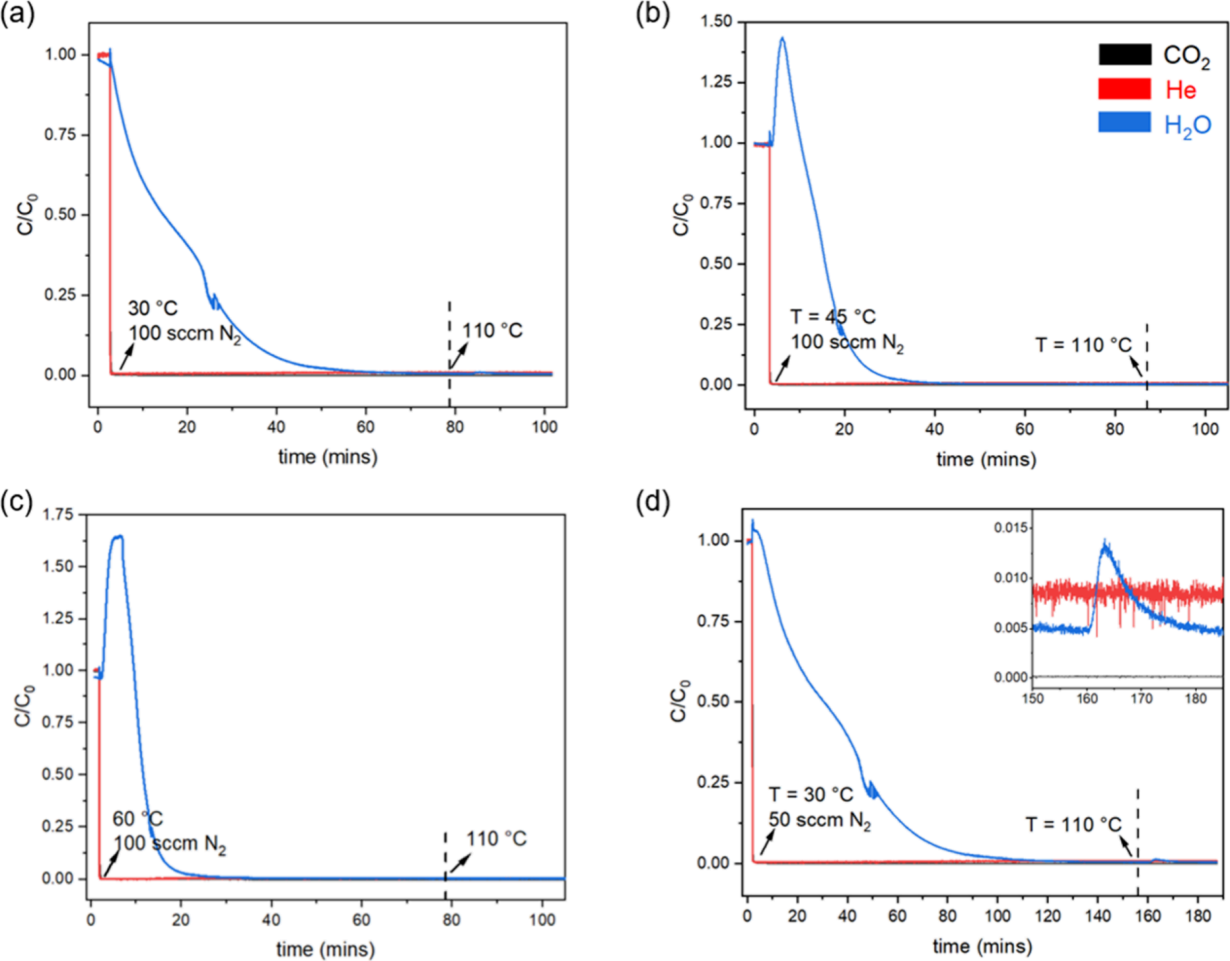
Desorption profiles of water post adsorption of humid flue gas (14% CO_2_, 70% RH, 30 °C) in a breakthrough experiment using (a) 100 sccm N_2_, 30 °C, (b) 100 sccm N_2_, 45 °C, (c) 100 sccm N_2_, 60 °C, and (d) 50 sccm N_2_, 30 °C. Dashed lines indicate the onset of heating the module, where the temperature is set to 110 °C. The inset in (d) shows some residual water desorption post heating.

The experimental data presented above provide a basis from which it would be possible to select process parameters for a practical SMB process. The actual choice of parameters on the industrial scale would depend on the trade-offs between capital costs (for bed lengths), feed processing requirements, and operational constraints such as heating/cooling times. Notably, with the use of the N_2_ raffinate as the desorbent, it is in principle possible to eliminate the requirement of any additional desorbent and the subsequent need to invest energy to dehumidify the desorbent for recycling. This approach would likely require some heat input to the bed in zone II for the complete desorption of water. The actual choice of desorption condition—flow rates and temperatures—can be determined in the future by comparing the energy required for desorbent dehumidification versus bed heating for the specific modules in consideration.

To further understand the potential use of the MOF in realistic separations, we investigated the stability of MUF-16 on exposure to SO_2_. Acid gases (SO_*x*_, NO_*x*_, H_2_S, etc.) are ubiquitous contaminants in flue gas, biogas, hydrocarbon separations, and other practical applications. Their presence can lead to significant degradation and reduction of the lifetime of adsorbents.^58^ Figure 7a shows the TGA uptake of CO_2_ (14% CO_2_, panel 1). The sample was activated at 150 °C for 3 h prior to measuring CO_2_ uptake. The CO_2_ was then desorbed by heating under nitrogen flow at 150 °C for 30 min, and the material was exposed to dry SO_2_ (200 ppm) for 12 h (panel 2). After desorbing SO_2_ for 30 min at 150 °C under nitrogen, the CO_2_ uptake was remeasured. We did not observe any reduction in uptake after exposure. The sample was characterized before and after exposure, and no signs of degradation were observed (SI, Section 6). Figure 7b shows results from cyclic adsorption–desorption of CO_2_ and SO_2_, with each cycle lasting 30 min. Throughout, we see consistent CO_2_ and SO_2_ uptakes, demonstrating complete desorption of SO_2_ from the MOF. However, prolonged exposure to humid SO_2_ streams can potentially lead to the formation and deposition of sulfurous acid in “wet” adsorbent beds, which will affect material stability differently and, therefore, warrants further investigation. The scope of this work is limited to testing exposure to SO_2_, so the observations may not necessarily extend to other acid gases such as NO_*x*_ or H_2_S.

**Figure 7:**
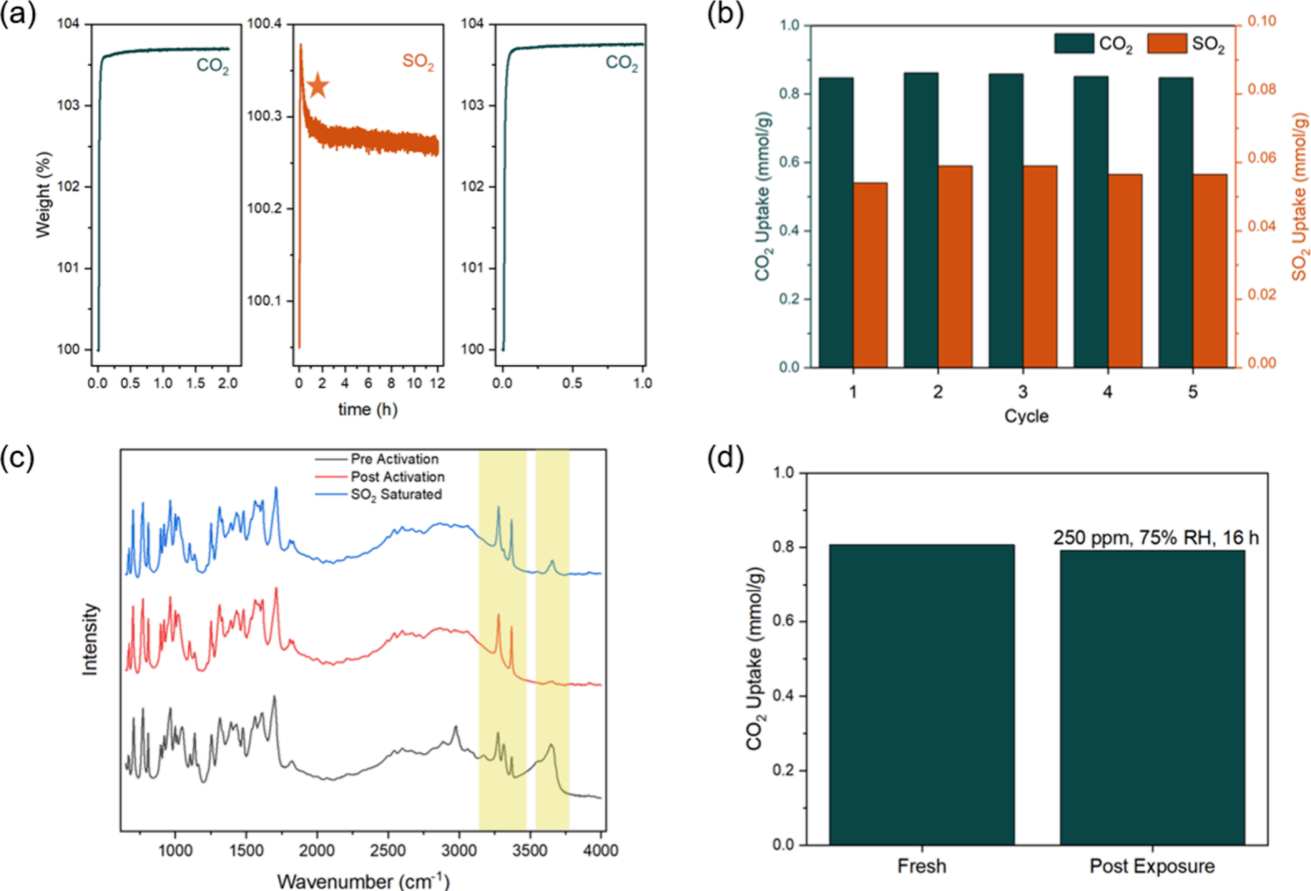
(a) CO_2_ (14 mol % concentration) uptake on a fresh, activated sample (left), SO_2_ uptake (middle), and CO_2_ uptake post exposure to dry SO_2_ (right) as measured on a TGA. The sudden increase in loading for SO_2_ uptake is an artifact of the switching of valves. (b) Cyclic adsorption of CO_2_ (green) and SO_2_ (orange) in 30 min sequential adsorption–desorption steps. (c) In situ DRIFTS spectra for MUF-16 pre (black) and post activation (red) and upon SO_2_ saturation (blue) at 30 °C. 1000 ppm of SO_2_ and balance N_2_ were used for this experiment. (d) CO_2_ uptake in MUF-16 at 14% CO_2_ concentration as measured on a TGA before and after exposure to humid SO_2_.

We also conducted in situ DRIFTS experiments to gain insight into the response of MUF-16 upon exposure to SO_2_. Figure 7c shows an IR spectrum of the MOF stored in a lab environment (where water adsorbed from ambient air is expected), activated in situ, and SO_2_ (1000 ppm) saturated. Comparing the SO_2_ saturated spectrum with the spectrum after activation, we observe a sharp band around 3648 cm^–1^ and a shoulder around 3317 cm^–1^ corresponding to O–H stretching and N–H stretching, respectively. These observations indicate that SO_2_ interacts with the MOF via weak H-bonding, consistent with the reversibility of the SO_2_ adsorption described above.

Finally, we also tested the stability of MUF-16 on exposure to humid SO_2_ for 4000 ppm hours at 75% RH, as described in Section 2.8. Examples are known of other MOFs that are stable upon exposure to dry acid gases but unstable upon exposure to humid acid gases.^47,50,59^ We assessed stability using standard characterizations (SI, Section 6) and measured the CO_2_ uptake before and after exposure. Figure 7d shows that there is no reduction of uptake of CO_2_ after exposure to humid SO_2_ (within experimental uncertainty), as also demonstrated previously by López-Cervantes et al.^58^

## 4 Conclusions

In this work, we proposed an SMB process for capturing CO_2_ from humid postcombustion flue gas using MUF-16 and demonstrated key attributes of this process experimentally. MUF-16 exhibits excellent selectivity for CO_2_ over nitrogen, but it does not adsorb any CO_2_ in the presence of humidity. The latter property may seem disadvantageous for the treatment of humid flue gas, but we showed that an SMB process can use this phenomenon productively. The SMB process takes advantage of lack of competitive sorption between CO_2_ and water and the relatively low heats of adsorption of CO_2_ (∼36 kJ/mol) and H_2_O (∼56 kJ/mol) in MUF-16 to obtain low overall energy requirements for humid flue gas capture of CO_2_. Other physisorbents typically have much higher H_2_O/CO_2_ sorption ratios and higher H_2_O sorption enthalpies. Sharp breakthrough curves for both CO_2_ and H_2_O observed for MUF-16 potentially facilitate process designs aimed at high product purities. The SMB configuration enables steady-state operation and uses the nitrogen raffinate as a desorbent for water, eliminating the need for a separate desiccant bed. The SMB approach also allows the desorption conditions for CO_2_ and H_2_O to be controlled independently. As a result, localized heating of the H_2_O saturated bed is possible, potentially reducing the overall energy costs.

Compared to a single-column fixed bed process, the use of localized heating, elimination of a desiccant bed, and reuse of N_2_ from the feed mixture as the desorbent stream substantially reduce the overall energy requirement of the process. Although we tested key attributes of the SMB process with experiments, a detailed quantification of energy savings from this process depends on the actual choice of module, desorption conditions (temperature and additional N_2_/air input), and cycle times. Subsequent analysis and process optimization will be needed to reliably quantify these savings and other essential parameters, such as CO_2_ recovery and process productivity.

We used experimental tests to establish that MUF-16 is robust with respect to exposure to SO_2_, a key contaminant of concern in flue gas. We did not, however, explore how the proposed SMB process would allow or prevent the accumulation of SO_2_ in the bed or in the desorbed CO_2_ stream. These potential issues are not specific to our proposed process; they are relevant to all flue gas treatment processes.

## Supplementary Material



## Notes

Breakthrough and humid exposure setup schematics; material characterizations (PXRD, FTIR-ATR, SEM); isotherm model parameters for experimental isotherms; CO_2_ breakthrough curves; and postexposure material characterization (PDF)
