# Supplementary material for: Simulated Moving Bed Process for CO2 Capture from Humid Postcombustion Flue Gases Using MUF-16
Source: ACS Appl Mater Interfaces. 2025 Oct 29;17(45):62122–30. doi: 10.1021/acsami.5c16139 (PMC12616598; doi:10.1021/acsami.5c16139)
Supplement: Supplementary file 1 [file am5c16139_si_001.pdf]

Supporting Information

Simulated Moving Bed Process for CO_2 Capture from Humid Post-combustion Flue Gases using MUF-16

Akriti Sarswat[1], Yoseph A. Guta[1], Mario Zorrilla-Valtierra[1], Anthony Cochran[1], Suhyun Kim[1], David S. Sholl[2]*, Ryan P. Lively[1]*

[1]School of Chemical & Biomolecular Engineering, Georgia Institute of Technology, Atlanta, GA, 30332-0100, USA

[2]Oak Ridge National Laboratory, Oak Ridge, TN 37830, USA

*shollds@ornl.gov, ryan.lively@chbe.gatech.edu

Table of Contents

	Section	Page no.
**1.**	Breakthrough Setup	3
**2.**	Humid Exposure Setup	4
**3.**	PXRD, FTIR-ATR and SEM	5
**4.**	Isotherm Model Parameters for Experimental Isotherms	6
**5.**	CO_2 breakthrough curves for MUF-16	8
**6.**	Post Exposure Characterization	10
**7.**	References	11

Section 1. Breakthrough Setup

Figure S1 shows the custom-built breakthrough set up used for single column breakthrough experiments throughout this work.

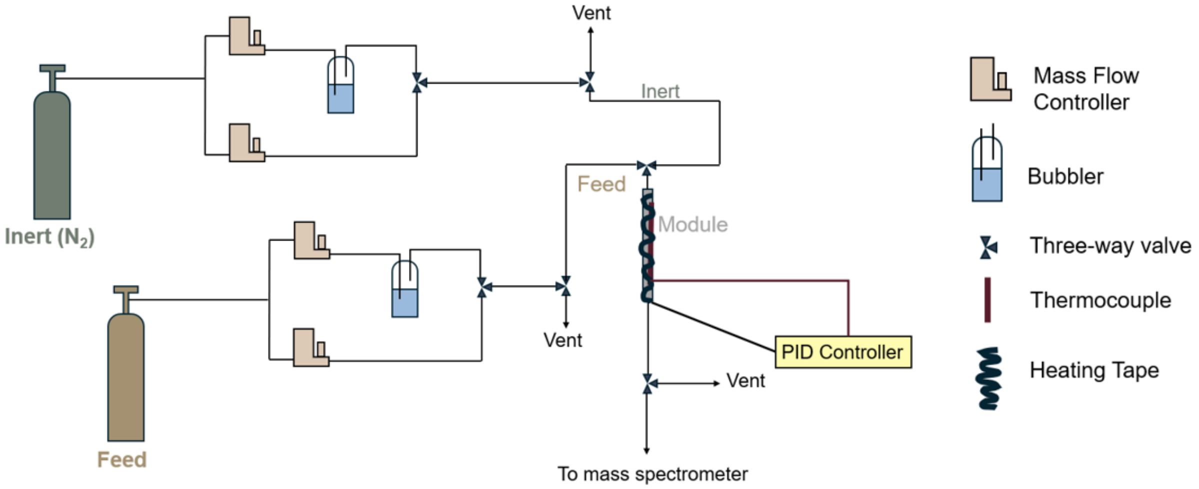

Figure S1: A schematic of the breakthrough set up used. Feed cylinder contains 14% CO_2, 2% He, balance N_2.

Section 2. Humid Exposure Setup

Figure S2 shows a custom-built setup for exposing samples to humid acid gases. The entire setup is equipped with SO_2 sensors and is placed in a well-ventilated chemical hood for safety.

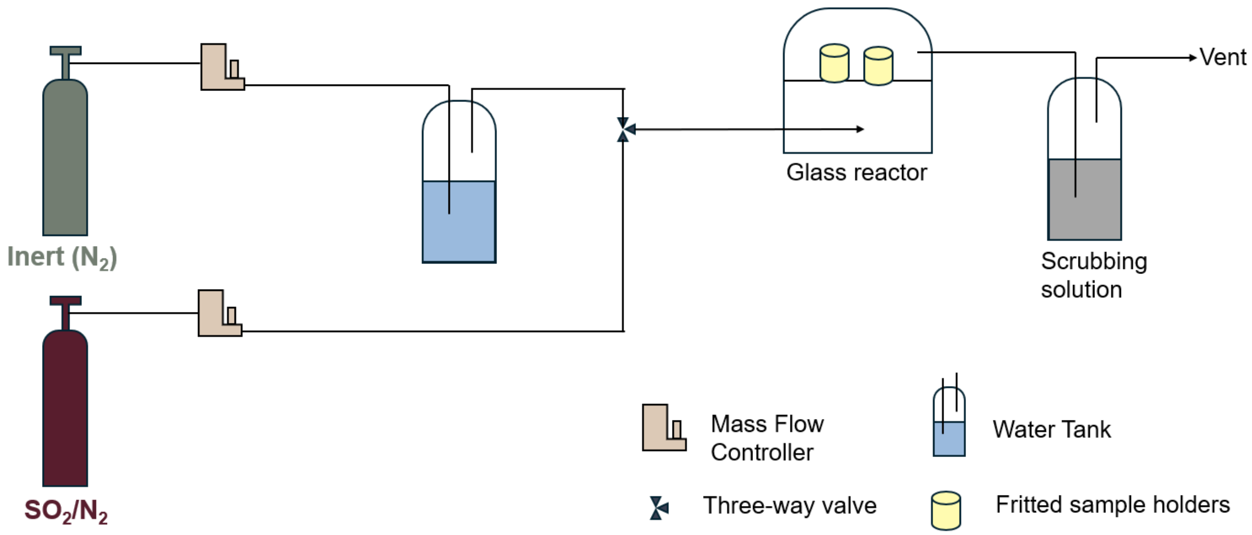

Figure S2: A schematic of the custom-built acid gas exposure setup used for exposing MUF-16 samples to humid SO_2

Section 3. PXRD, FTIR-ATR and SEM for fresh samples

PXRD patterns for MUF-16 (post activation) are in agreement with the patterns reported in literature (Figure S3).[1-3] SEM shows sheet like crystals for fresh MUF-16 synthesis, indicating successful synthesis, in line with our previous observations.[1]

Our previous work on the synthesis of MUF-16 showed that FTIR-ATR was needed in addition to SEM and PXRD to confirm successful synthesis.[1] FTIR-ATR spectra recorded ex-situ for fresh MOF is shown in Figure S3 (c).

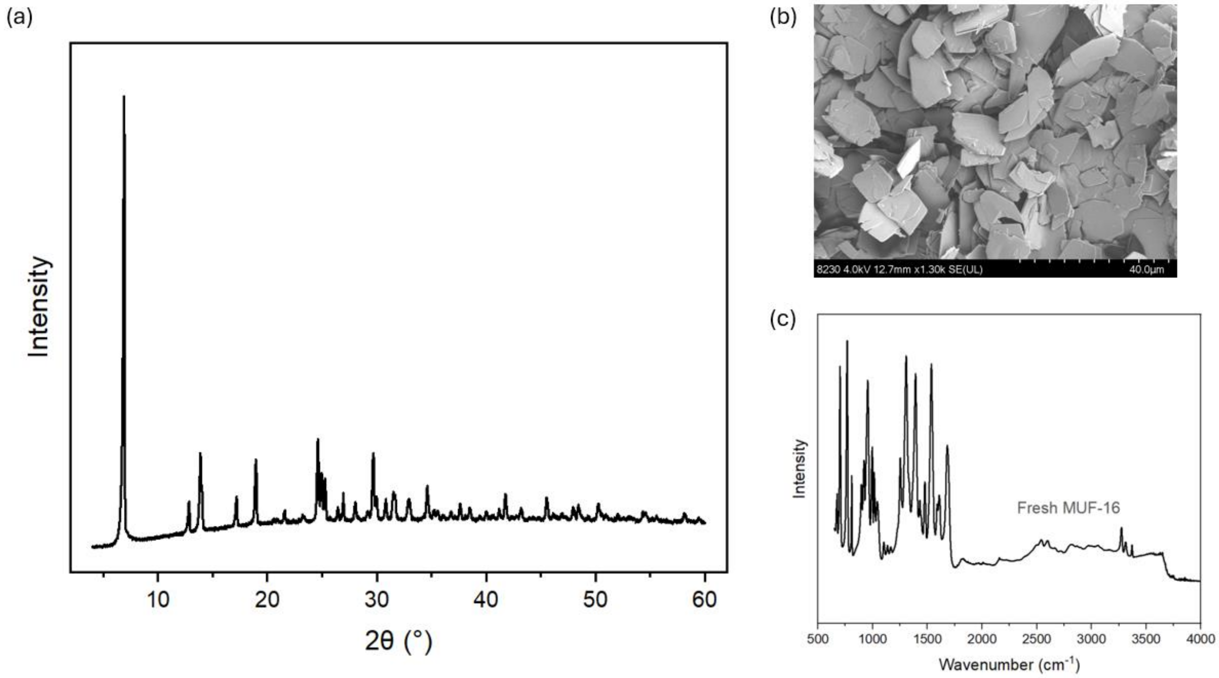

Figure S3: Characterizations for (post activation) MUF-16 (a) PXRD (b) SEM (c) ATR-FTIR

Section 4. Dual-site Langmuir Freundlich Isotherm Model Parameters for Experimental Isotherms

Isotherms for CO_2 and water were recorded at 3 different temperatures to determine heats of adsorption using the Clausius - Clapeyron equation. The CO_2 isotherms were fitted to the Langmuir model given by

$q_i(p_i) = q_i^{sat} \frac{k_i p_i}{1+ k_i p_i}$, where q_i is the uptake at pressure p_i , q_i^{sat} is the saturation loading at the given temperature and k_i is a fitting parameter. q_i^{sat} and k_i are obtained by fitting experimental isotherm data to the equation.

The H_2O isotherms were fitted to the dual site Langmuir Freundlich Model given by

$q_i(p_i) = q_{1_i} \frac{(k_{1_i} p_i)^{n_{1_i}}}{1+(k_{1_i} p_i)^{n_{1_i}}} + q_{2_i} \frac{(k_{2_i} p_i)^{n_{2_i}}}{1+(k_{2_i} p_i)^{n_{2_i}}}$, where q_i is the uptake at pressure p_i. $q_{1_i}, q_{2_i}, k_{1_i}, k_{2_i}, n_{1_i}, n_{2_i}$ are obtained by fitting experimental isotherm data to the equation.

Figure S4 and S5 show experimental isotherms and Langmuir-Freundlich model fitting for CO_2 and H_2O at three different temperatures. The fitted parameters are given in table S1.

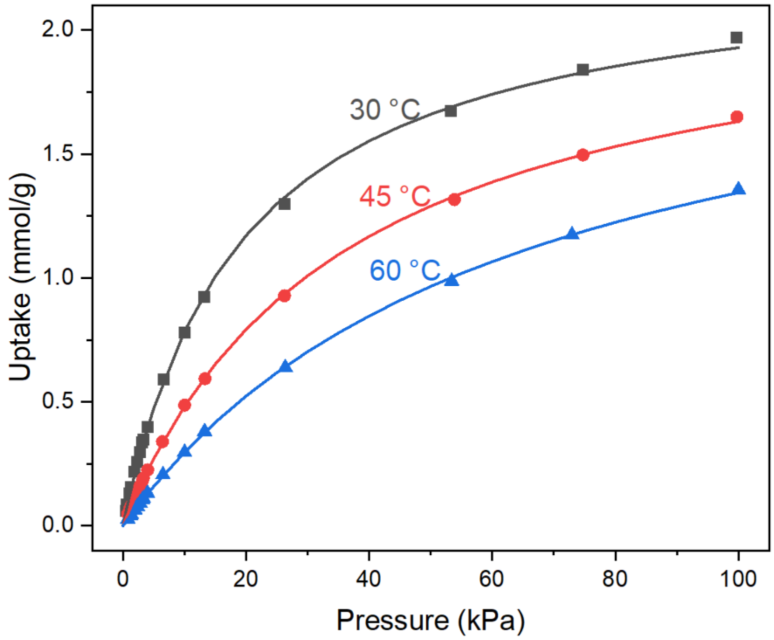

Figure S4: Experimental data (symbols) vs Langmuir model fitting (line) for CO_2 at 30 °C (black), 45 °C (red) and 60 °C (blue).

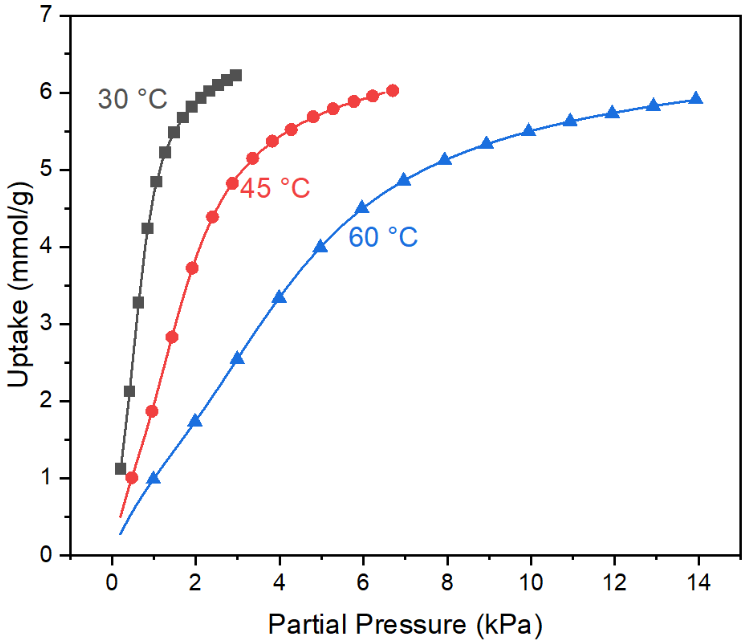

Figure S5: Experimental data (symbols) vs Langmuir-Freundlich model fitting (line) for H_2O at 30 °C (black), 45 °C (red) and 60 °C (blue).

Table S1: (a) Langmuir Model Parameters for CO_2 (b) Langmuir-Freundlich Model Parameters for H_2O

(a)

	CO_2		
Parameter	**30 °C**	**45 °C**	**60 °C**
q_i (mmol/g)	2.300	2.217	2.209
k_i (kPa^{-1})	0.0519	0.027	0.016

(b)

		H_2O	
Parameter	**30 °C**	**45 °C**	**60 °C**
q_{1_i} (mmol/g)	2.236	4.639	2.513
k_{1_i} (kPa^{-1})	1.446	0.505	0.234
n_{1_i}	3.798	0.927	3.086
q_{2_i} (mmol/g)	4.860	2.555	4.773
k_{2_i} (kPa^{-1})	1.465	0.565	0.213
n_{2_i}	1.043	3.231	0.886

Section 5. CO_2 Breakthrough Curves for MUF-16

Breakthrough curves measured with a flowrate of 50 sccm showed a minor roll up for CO_2 (Figure 3 of main manuscript). We hypothesize that the roll up is due to re-caliberation of pressure and composition in the bed. At a slower feed flow rate of 15 sccm, neglegible roll up is observed as shown in Figure S6.

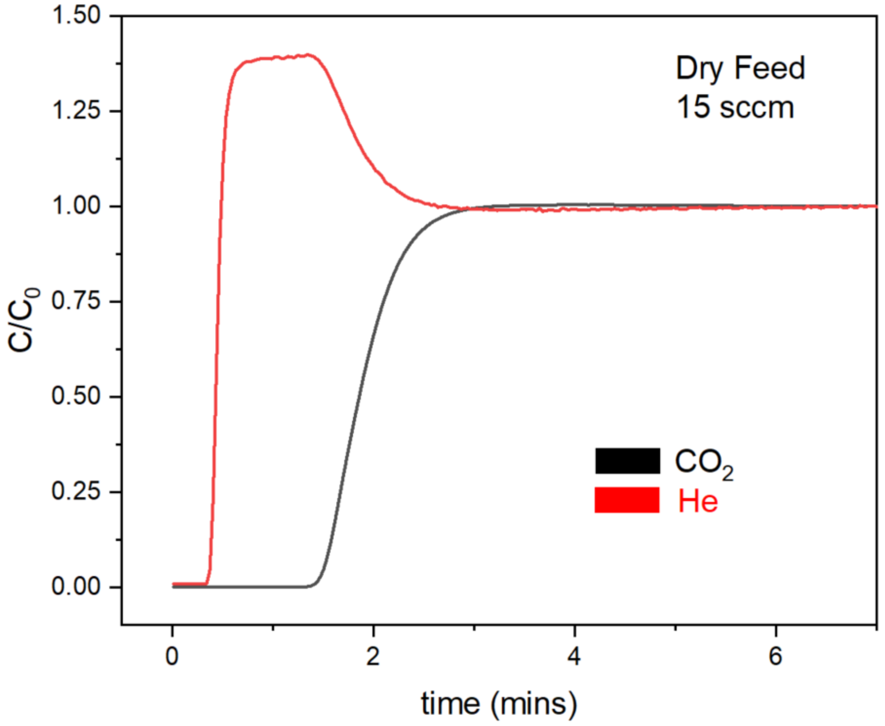

Figure S6: CO_2 breakthrough curves for MUF-16 with dry 14% CO_2 feed at 30 ºC with a flow rate of 15 sccm. He (2%) was used as a tracer. Breakthrough of He indicated onset of adsorption.

Total uptake amounts can obtained by integrating the area under the breakthrough curve as shown in Figure S7.

$CO_2\ adsorbed = \frac{(Area\ I - Area\ II) * Flow\ rate\ (sscm) * CO_2\ feed\ concentration}{mass\ of\ sample\ (g) * 22.4 \frac{cc}{mol}}$ = 0.9 mmol/g which is in agreement with single component isotherm results.

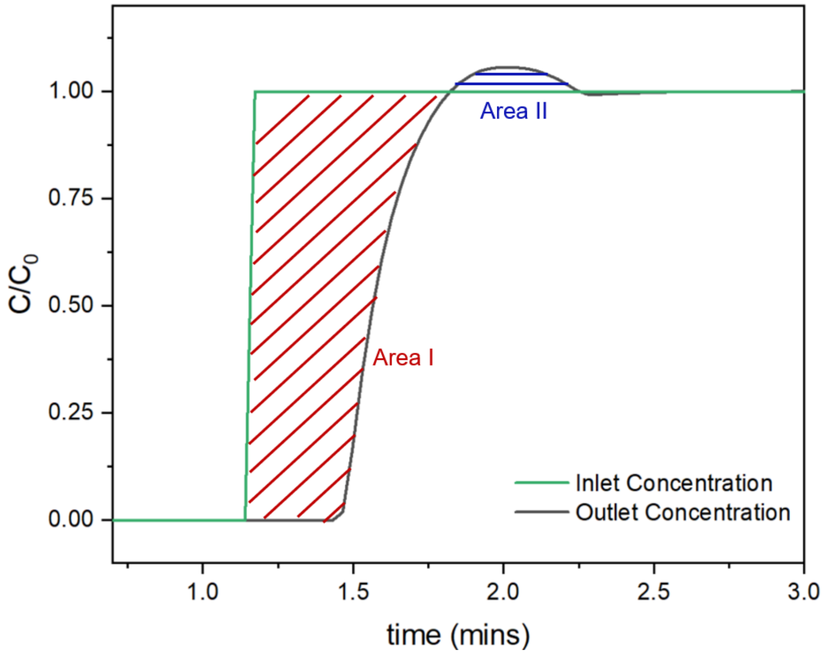

Figure S7: CO_2 breakthrough curves for MUF-16 with dry 14% CO_2 feed at 30 ºC with a flow rate of 50 sccm (black), also shown in Figure 4(a) in the main manuscript. Inlet concentration is indicated in green. Adsorbed CO_2 is indicated by the difference in area I and area II.

Section 6. Post Exposure Characterization

PXRD patterns for fresh MOF and on exposure to dry and humid SO_2 are shown in Figure S3. The pattern for fresh MOF is in good agreement with literature.[1-3] No signs of degradation are observed from the PXRD patterns measured post long exposures to dry and humid SO_2.

No signs of etching or degradation are observed on exposure to dry or humid SO_2.

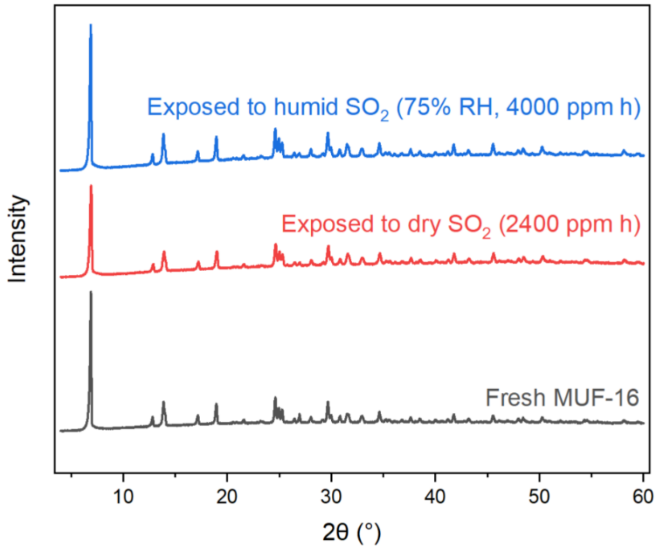

Figure S8: PXRD patters for fresh MOF (grey), MOF exposed to 200 ppm dry SO_2 for 12 hours (red), MOF exposed to 250 ppm humid SO_2 for 16 hours at 75% RH (blue).

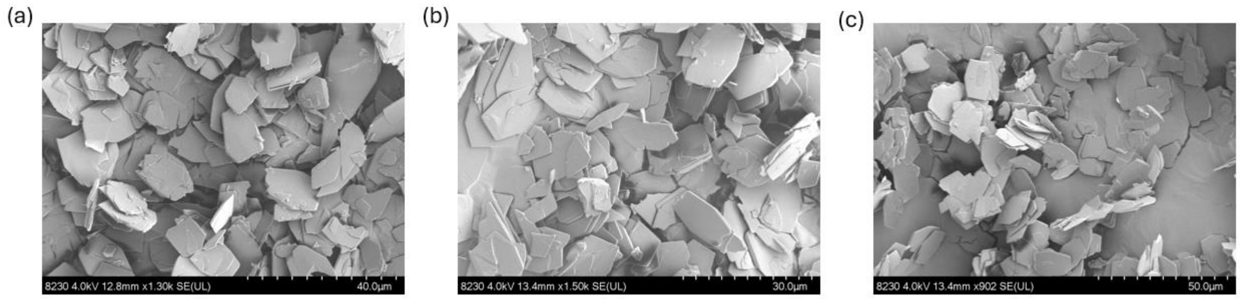

Figure S9: SEM images for (a) fresh MUF-16 (b) MOF exposed to 200 ppm dry SO_2 for 12 hours (c) MOF exposed to 250 ppm humid SO_2 for 16 hours at 75% RH.

Section 7. References

1. Sarswat, A., et al., *Investigating the Effect of Trace Levels of Manganese Ions During Solvothermal Synthesis of Massey University Framework-16 on CO_2 Uptake Capacity.* Chemistry of Materials, 2024. **36**(11): p. 5378-5387.
2. Qazvini, O.T. and S.G. Telfer, *MUF-16: A Robust Metal–Organic Framework for Pre- and Post-Combustion Carbon Dioxide Capture.* ACS Applied Materials & Interfaces, 2021. **13**(10): p. 12141-12148.
3. Qazvini, O.T., R. Babarao, and S.G. Telfer, *Selective capture of carbon dioxide from hydrocarbons using a metal-organic framework.* Nature Communications, 2021. **12**(1): p. 197.
